# Contribution to the subfamily Panchaetothripinae (Thysanoptera, Thripidae) from Saudi Arabia, with two new genera records and an illustrated key

**DOI:** 10.3897/BDJ.13.e175847

**Published:** 2025-11-20

**Authors:** Iftekhar Rasool, Amin N Al-Ansi, Hathal Mohammed Al Dhafer

**Affiliations:** 1 King Saud University Museum of Arthropods (KSMA), Department of Plant Protection, College of Food and Agriculture Sciences, King Saud University, P.O. Box 2460, Riyadh 11451, Saudi Arabia King Saud University Museum of Arthropods (KSMA), Department of Plant Protection, College of Food and Agriculture Sciences, King Saud University, P.O. Box 2460 Riyadh 11451 Saudi Arabia; 2 King Saud University, College of Food and Agriculture Sciences, Riyadh, Saudi Arabia King Saud University, College of Food and Agriculture Sciences Riyadh Saudi Arabia

**Keywords:** Arabian Peninsula, checklist, distribution, *
Hercinothrips
*, new records, *
Rhipiphorothrips
*

## Abstract

**Background:**

The subfamily Panchaetothripinae Bagnall (Thysanoptera, Thripidae) comprises about 150 species belonging to 42 genera worldwide. Amongst these, twelve species belonging to six genera were previously recorded from the Arabian Peninsula, including eight species in four genera from Saudi Arabia. The Faunistic studies on the subfamily have been confined to the tropical and subtropical parts of Africa, America, Southeast Asia and Australia. However, the tropical regions of the Arabian Peninsula remained under-studied, with no considerable work having been done on this subfamily.

**New information:**

We present the first illustrated key to the Panchaetothripinae genera of the Arabian Peninsula and a checklist of Arabian species to facilitate their recognition. Moreover, two genera and species, *Hercinothrips femoralis* (Reuter) and *Rhipiphorothrips cruentatus* Hood, are recorded as new to Saudi Arabia. New distributional data for each species is also given. The presence of greenhouse thrips *Heliothrips haemorrhoidalis* Bouche in the country is doubtful. The species has not been recovered despite extensive surveys across the country spanning over six years. However, it is kept in the Arabian species list until further confirmations.

## Introduction

The subfamily Panchaethripinae comprises about 150 species belonging to 42 genera (ThripsWiki 2025). The species are predominantly distributed across tropical regions of the world. The majority of species are native to the Old-World tropics, in Africa and Southeast Asia; however, a small number are endemic to Australia and South America (Mound 2009). In the Arabian Peninsula, twelve species belonging to six genera have been previously recorded (Table 1), eight of these species in four genera being recorded from Saudi Arabia (Rasool et al. 2023). All of these species have been collected from the southern part of the Peninsula (Oman, southwest of Saudi Arabia, United Arab Emirates and Yemen) (zur Strassen 1990, Mirab-balou 2018, Rasool et al. 2023), which is an Afrotropical region in nature, supported by several studies (Larsen 1984, Hölzel 1998, Abdel-Dayem et al. 2018). However, none of these species is known from Bahrain, Kuwait and Qatar that are situated to the northeast of the Peninsula. The distributional trend of the Panchaethripinae suggests that the southwest area of Saudi Arabia harbours notable diversity in this subfamily with further exploration needed in the region. The presence of greenhouse thrips *Heliothrips haemorrhoidalis* Bouche in the country is doubtful, as that was enlisted by Martin (1972) without any substantial details. The species has not been recovered despite the extensive surveys conducted in all kinds of natural and agroecosystems and greenhouses during 2019–2025.

Species of the Panchaethripinae can be recognised amongst other subfamilies of Thripidae by a strongly reticulated head and body; long and needle-like terminal antennal segment, III–IV usually with long apical neck; first vein of fore-wing almost fused with coastal vein near base; meso and metasternal furca transverse, without median spinula (Masumoto 2010). The objective of this article is to present an illustrated diagnostic key to Panchaethripinae genera, a checklist to the Arabian species and to record *Hercinothrips* Bagnall and *Rhipiphorothrips* Morgan for the first time from Saudi Arabia.

## Materials and methods

The current study is based on fresh collections of thrips from the southwest provinces of Saudi Arabia using beating sheets (BT). Collected specimens were preserved in 80–95% ethanol. Slides were prepared by using stereomicroscope from Meiji Techno EMZ–10 Series 7X–45X Zoom. The images were produced by using an automontage software system (Syncroscopy, Cambridge, UK), attached to a phase contrast microscope (DM2500, Leica®, Germany).

### Abbreviations

**KSMA**: King Saud University Museum of Arthropods, Riyadh, Saudi Arabia. **BT**: Beating sheet.

## Taxon treatments

### Hercinothrips

Bagnall, 1932

32B91F65-981F-595B-A85B-203C6A0EDC55

Hercinothrips Bagnall, 1932: 506. Type species: *Heliothrips bicinctus* Bagnall 1919. Type locality: Belgium.

#### Taxon discussion

The genus *Hercinothrips* is an old-world genus that consists of ten species worldwide. All of the species are described from Africa, except *H. splendens* Goldaracena and Vierbergen from Netherlands, *H. femoralis* (Reuter) from Finland and *H. bicinctus* (Bagnall) from Belgium (ThripsWiki 2025). The latter two species are widespread in the tropical and subtropical countries, the first of these being a pest particularly in greenhouses and the second has been recorded as damaging banana plants (Moritz et al. 2013, Mound and Wells 2015). In the Arabian Peninsula, the two species, *H. femoralis* and *H. tenuis* Hartwig, are known from Yemen (zur Strassen 1990); however, the genus is recorded for the first time from Saudi Arabia. The species of this genus are unusual amongst Panchaetothripinae genera in having both veins of fore-wings with a complete row of setae (Lima et al. 2020). The other characters are 8-segmented antennae, with segments III and IV bearing forked sense cones; 2-segmented tarsi (Fig. 2f); head with weak transverse ridge posteriorly, visible as a small projection laterally (Fig. 3b).

### Hercinothrips femoralis

(Reuter, 1891)

74127CD7-998E-502F-BD54-E07671266C02

Heliothrips femoralis Reuter, 1891: 166. Type locality: Finland.

#### Materials

- **Type status:** Other material. **Occurrence:** recordedBy: Rasool, I.; individualCount: 1 ♀; lifeStage: adult; occurrenceID: EE66A9EE-1F2E-566A-82F0-C0B8CBDC4F12; **Location:** country: Saudi Arabia; stateProvince: Jazan; locality: Al Aridha, Sala Mountains, Wadi Rough; verbatimElevation: 631 m; verbatimLatitude: 17°01'52.2; verbatimLongitude: 43°08'16.5; **Event:** samplingEffort: BT; eventDate: 23/2/2024

#### Diagnosis

Fully mature female with brown body (Fig. 5a); head with two longitudinal yellow areas or yellowish anteriorly with posterior brown area (Fig. 3b); antennal segment II and VI–VIII brown, III–V yellow, except IV and V light brown at apex (Fig. 1b); pronotum with variably light brown spots; legs yellow with mid and hind femora brown; fore-wings with sub-basal area and apex pale, with median area variably brown (Fig. 2b). The species is very close to *H. bicinctus* in general appearance and features; however, it can be distinguished by: antennal segments VI–VIII brown (Fig. 1b); median pale area of fore-wings absent (Fig. 2b). In contrast, in *H. bicinctus*, antennal segments VI–VIII are yellow and median pale area of fore-wings about 2.0 times as long as first dark cross band (Goldaracena and Vierbergen 2022).

#### Distribution

The species was described from Finland and is now widespread across North, Central and South America, throughout Africa, Europe, tropical and subtropical Asia, Australia and New Zealand (Moritz et al. 2013).

### Rhipiphorothrip

Morgan, 1913

D16E8F15-C0F6-5354-B497-736A274C07A0

Rhipiphorothrips Morgan, 1913: 17. Type species: *Rhipiphorothrips pulchellus* Morgan, 1913. Type locality: Sri Lanka.

#### Taxon discussion

The *Rhipiphorothrips* is an Old World palaeotropical genus, comprising five species. Two of these are known from tropical Africa and three from tropical Asia and China (Wilson 1975, ThripsWiki 2025). In the Arabian Peninsula, the genus was known only from Oman (Mirab-balou 2018) and is recorded for the first time from Saudi Arabia. The members of the genus are unique amongst other Panchaetothripinae genera by having strongly rugose sculpture on head, pronotum and body surface. It is close to *Australothrips* Bagnall, *Phibalothrips* Hood and *Retithrips* Marchal in lacking fringed cilia on the fore-wing anterior margins. However, these genera have polygonal reticles on the head instead of rugose sculptures as in *Rhipiphorothrips* (Wilson 1975). The other important characters of the genus include: 8-segmented antennae, without microtrichia, segments III–IV with simple (*Rh. cruentatus* and *Rh. pulchellus*) or forked (*Rh. africanus*, *Rh. concoloratus* and *Rh. miemsae*) sense cones (Wilson 1975, Zhang and Tong 1993); fore-wings anterior margin without cilia cf (Fig. 2c); tarsi 1-segmented; mesonotum completely divided medially with a longitudinal suture (Fig. 3f); metanotum raised, sculptured, in shape of inverted triangle (Fig. 3f); abdominal tergites III–IX with rugose sculpture laterally, each with median depression and polygonal reticules medially and comb-like microtrichia at anterior margin (Fig. 4b); tergites IX and X with setae on hind margin short and fan-shaped at apices (Fig. 4d).

### Rhipiphorothrips cruentatus

Hood, 1919

01753722-5D92-55BE-95E8-C3558ABA0C4A

Rhipiphorothrips cruentatus Hood, 1919: 94. Type locality: India.

#### Materials

- **Type status:** Other material. **Occurrence:** recordedBy: Rasool, I.; individualCount: 6♀2♂; lifeStage: adult; occurrenceID: 40427E8B-02AE-5BDF-9383-C8856D9E906F; **Location:** country: Saudi Arabia; stateProvince: Tabouk; locality: Umluj; verbatimElevation: 57 m; verbatimLatitude: 25°04'03.0; verbatimLongitude: 37°17'31.3; **Event:** samplingEffort: SW; eventDate: 9/2/2022

#### Diagnosis

Female dark brown (Fig. 5b); antennae and legs yellow, antennal segment VI at apex and VII–VIII light brown (Fig. 1e); fore-wings pale with light brown base. Male strongly bicoloured with head, pronotum meso and metanotum dark brown, antennae, abdomen and legs yellow (Fig. 5c). This species is close to *Rh. pulchellus* in general features and sharing the simple sense cone on antennal segments III–IV (Fig. 1e). However, it can be identified by the dark brown female and bicoloured male; male with small, circular pore plate at anteromedian margin of sternites III–VII and is unique in having a prominent tooth-like lateral processes on abdominal segment IV (Fig. 4e). In contrast, *Rh. pulchellus* female is characterised by yellow pronotum and male lacks tooth-like lateral process on abdominal segment IV (Wilson 1975).

#### Distribution

The species was described from India, where it is widespread in most of the Indian States. It is also known from Afghanistan and west Pakistan (Wilson 1975), Bangladesh, Burma, China, Iran, Myanmar, Sri Lanka, in Arabian Peninsula from Oman (Mirab-balou 2018) and is new to Saudi Arabia.

## Identification Keys

### Key to the Panchaetothripinae genera from Arabian Peninsula

**Table:** 

1	Fore-wings anterior margin without cilia (Fig. 2c, d)	2
–	Fore-wings anterior margin with cilia (Fig. 2a, b)	4
2	Fore-wings broad, anterior margin with three protruding callosities (Fig. 2d); antennal segments III–IV, each with forked sense cones	*** Retithrips ***
–	Fore-wings long, slender, parallel-sided, without callosities or tubercles (Fig. 2c); antennal segments III–IV, each with simple sense cones (Fig. 1c, e) (rarely forked)	3
3	Antennae 7-segmented (Fig. 1c); head with polygamical sculptures (Fig. 3c); fore-wings second vein without setae; mesonotum entire, not divided medially (Fig. 3e); abdominal tergites II–VII smooth medially, with median lobate longitudinal ridge (Fig. 4a); setae S1 on abdominal tergites IX–X with pointed apex (Fig. 4c)	*** Phibalothrips ***
–	Antennae 8-segmented (Fig. 1e); head with rugose sculptures (Fig. 3d); fore-wings second vein with setal row complete; mesonotum divided medially with a longitudinal suture (Fig. 3f); abdominal tergites sculptured medially, without any lobate ridge (Fig. 4b); setae S1 on abdominal tergites IX–X with dilated apex (Fig. 4d)	*** Rhipiphorothrips ***
4	Tarsi 2-segmented (Fig. 2f); both fore-wing veins with complete row of setae (Fig. 2b); head with weak occipital ridge, a small projection visible posterolateral (Fig. 3b)	*** Hercinothrips ***
–	Tarsi 1-segmented (Fig. 2e); fore-wings vein with setal rows widely spaced and incomplete (Fig. 2a); head without occipital ridge (Fig. 3a)	5
5	Head and pronotum sculptures smooth, without internal markings; antennal segments III–IV each with simple sense cones; metanotum with prominent sculptured triangle; hind coxae simple	Heliothrips*
–	Head and pronotum sculptures with internal markings (Fig. 3a); antennal segments III–IV each with forked sense cones (Fig. 1a); metanotum normal, without triangle; hind coxae with prominent coiled internal apodeme	*** Caliothrips ***

[Heliothrips* not examined, adapted from Silva et al. (2024)]

## Supplementary Material

XML Treatment for Hercinothrips

XML Treatment for Hercinothrips femoralis

XML Treatment for Rhipiphorothrip

XML Treatment for Rhipiphorothrips cruentatus

## Figures and Tables

**Figure 1a.**
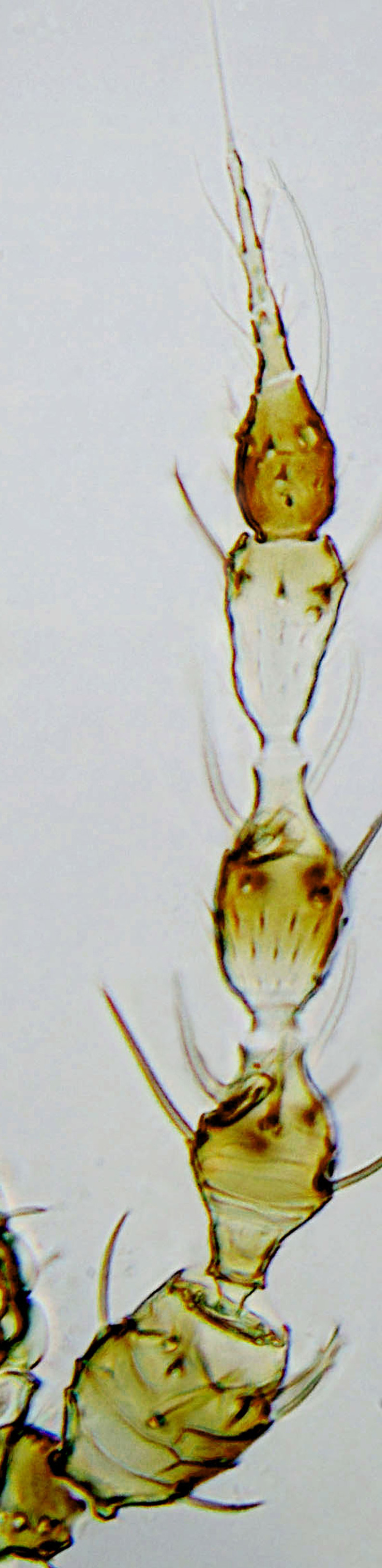
*Caliothrips helini* Hood;

**Figure 1b.**
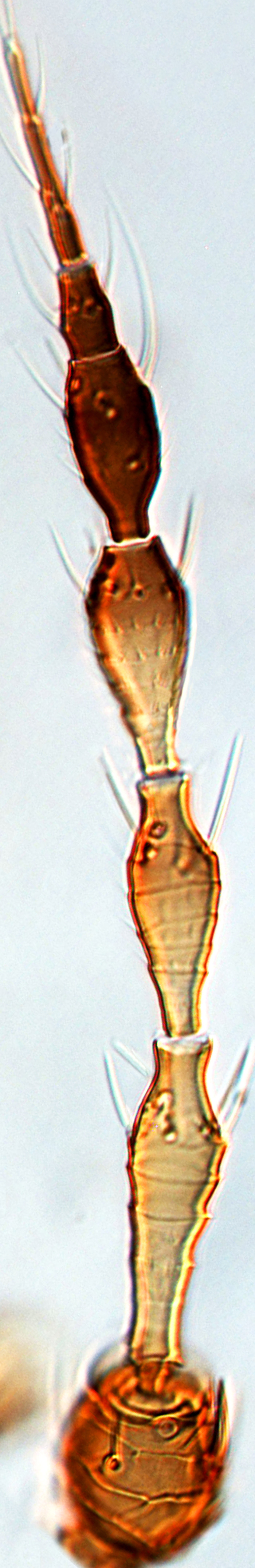
*Hercinothrips femoralis* Reuter;

**Figure 1c.**
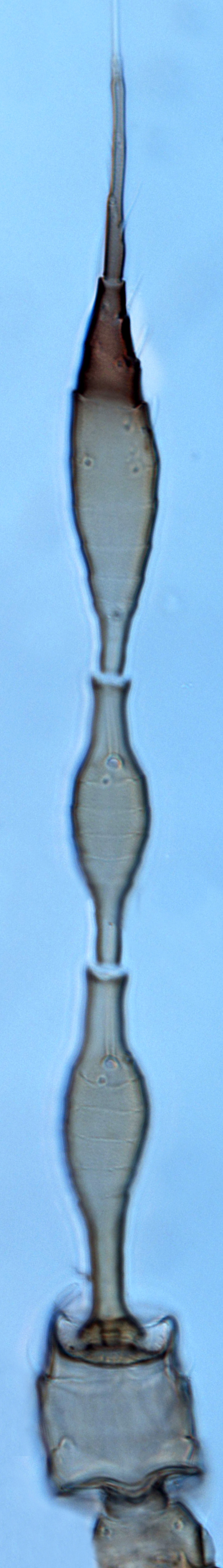
*Phibalothrips peringueyi* (Faure);

**Figure 1d.**
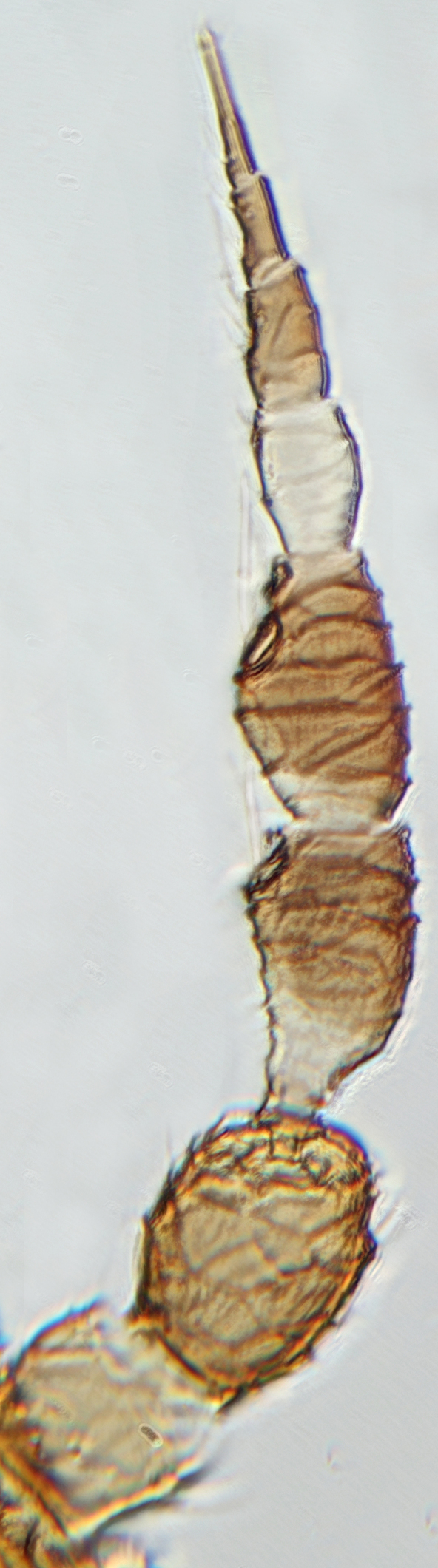
*Retithrips syriacus* (Mayet);

**Figure 1e.**
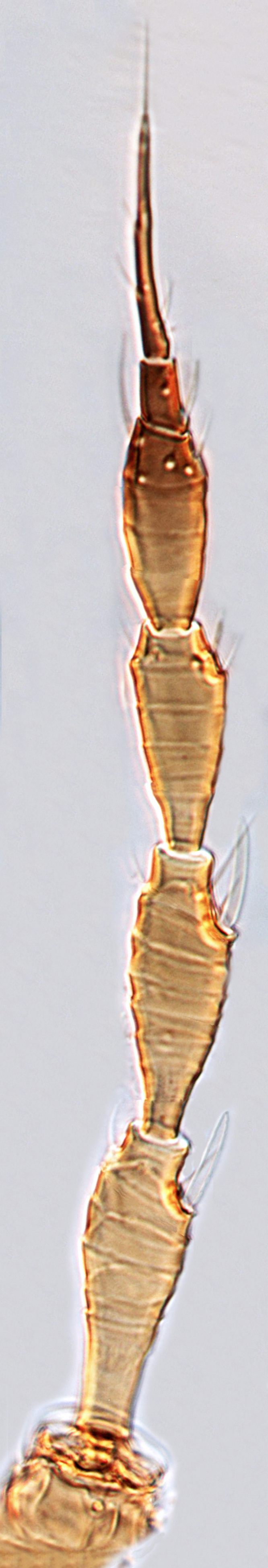
*Rhipiphorothrips cruentatus* Hood.

**Figure 2a.**
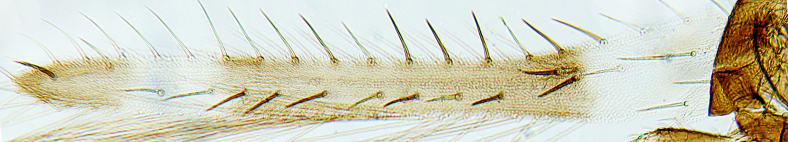
*Caliothrips helini* Hood;

**Figure 2b.**
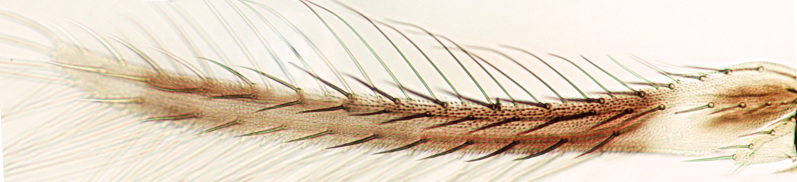
*Hercinothrips femoralis* Reuter;

**Figure 2c.**
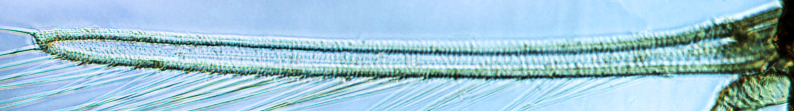
*Phibalothrips peringueyi* (Faure);

**Figure 2d.**
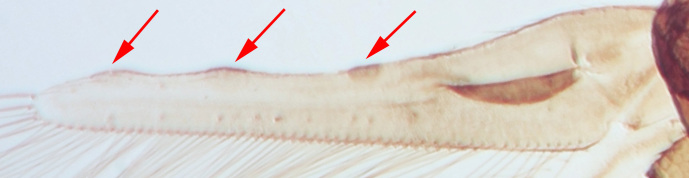
*Retithrips syriacus* (Mayet);

**Figure 2e.**
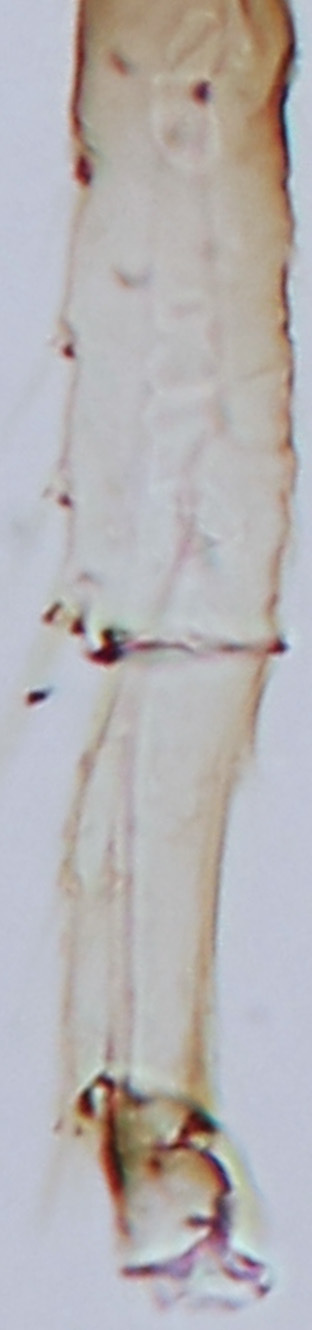
*Caliothrips luckmanni* Wilson;

**Figure 2f.**
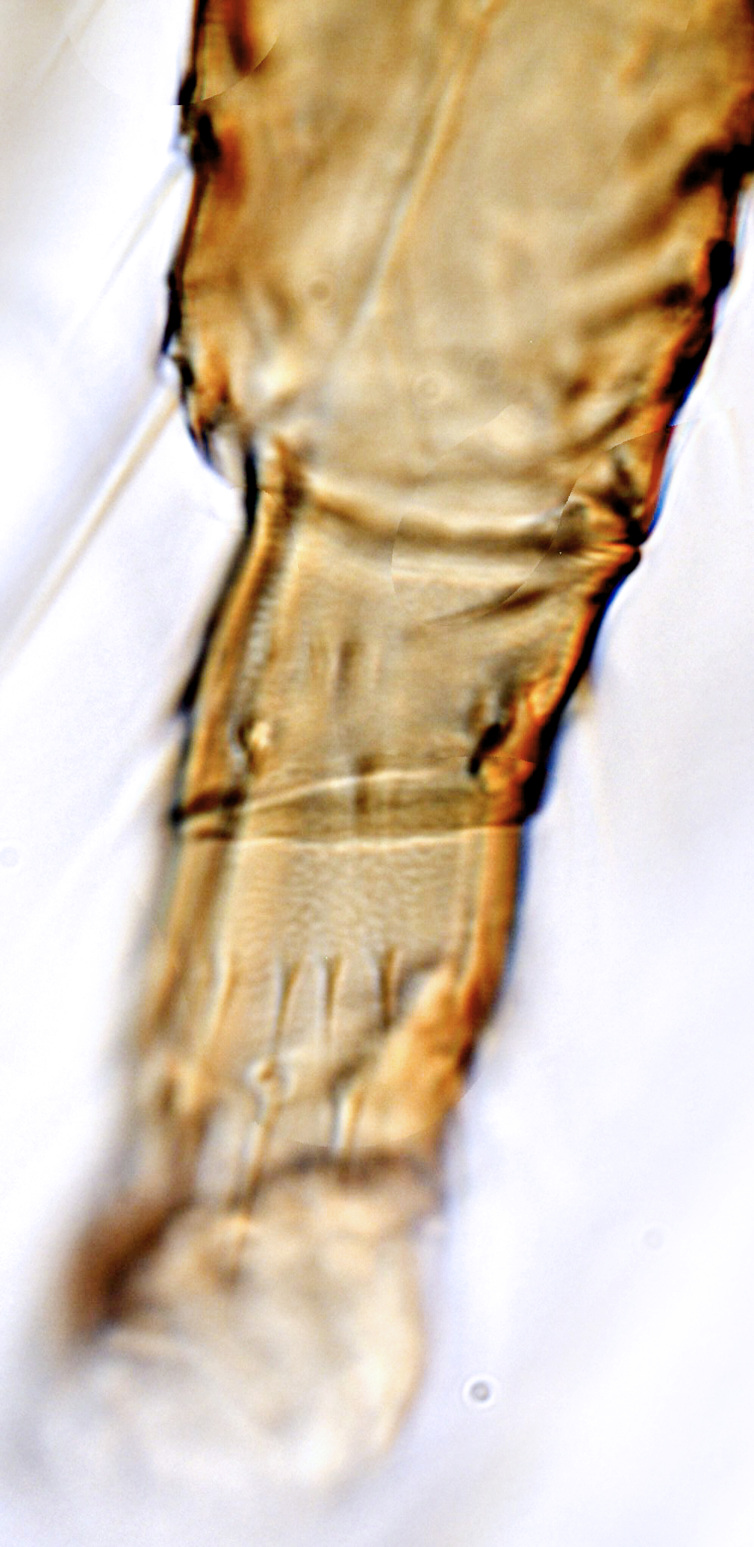
*Hercinothrips femoralis* Reuter.

**Figure 3a.**
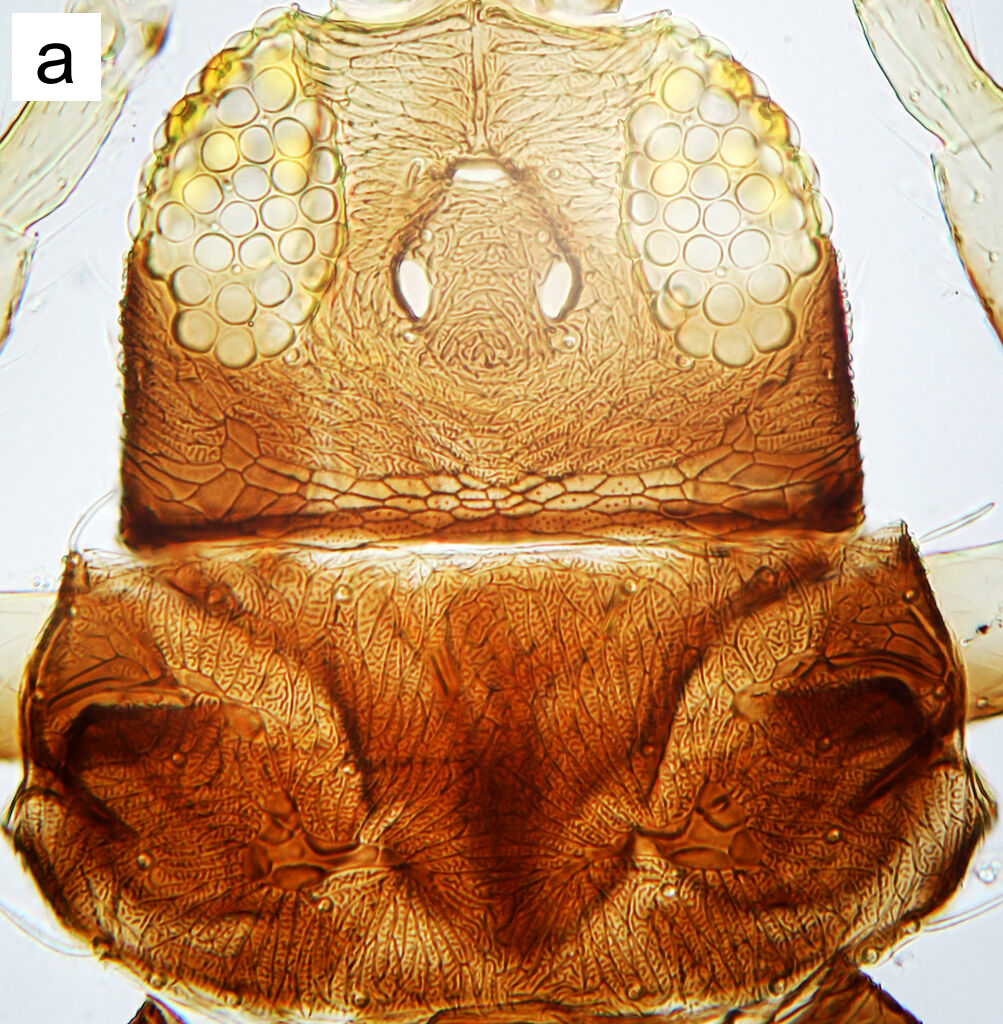
*Caliothrips quadrifasciatus* (Girault);

**Figure 3b.**
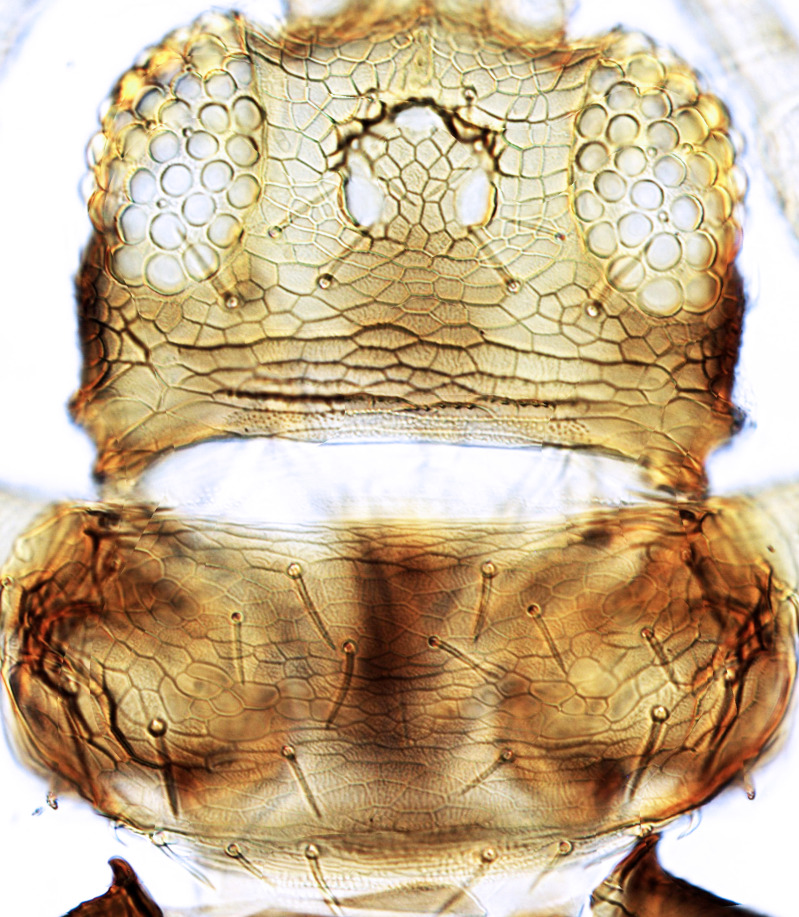
*Hercinothrips femoralis* Reuter;

**Figure 3c.**
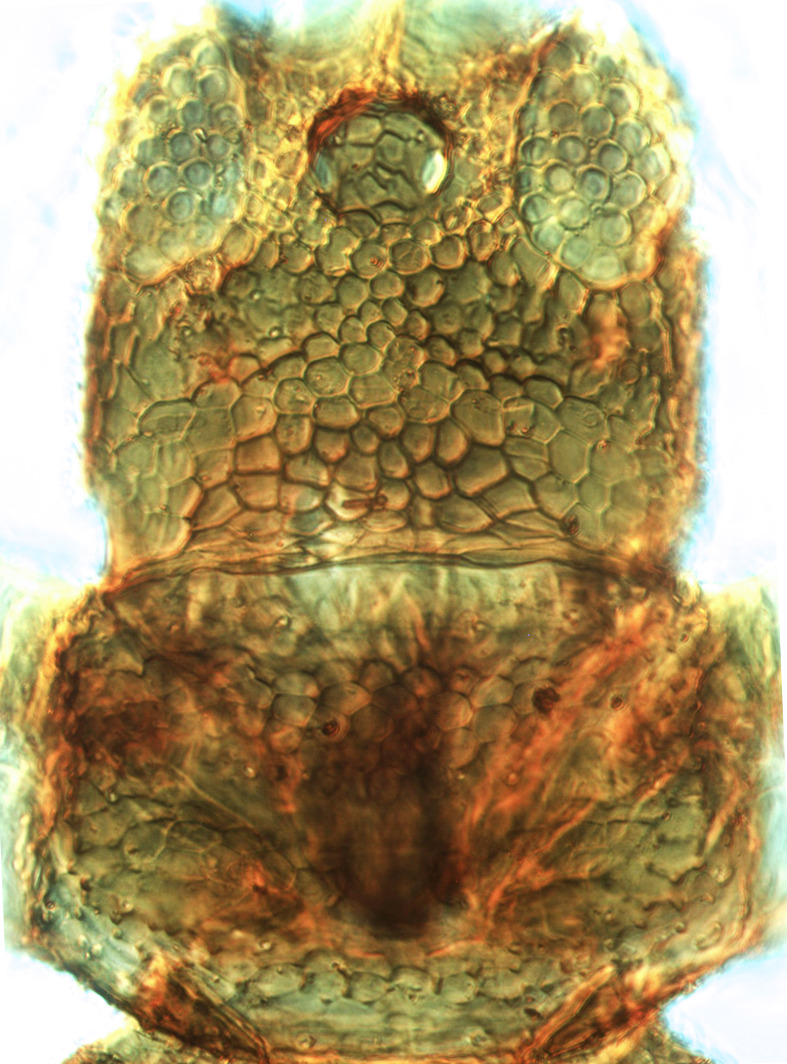
*Phibalothrips peringueyi* (Faure);

**Figure 3d.**
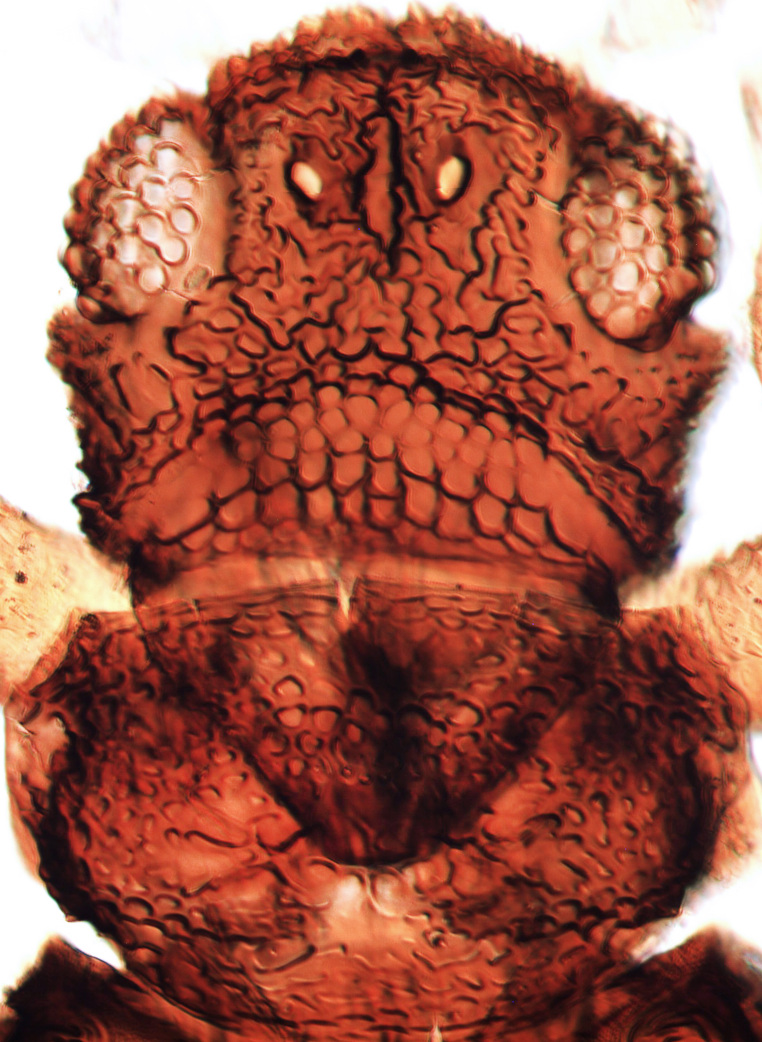
*Rhipiphorothrips cruentatus* Hood;

**Figure 3e.**
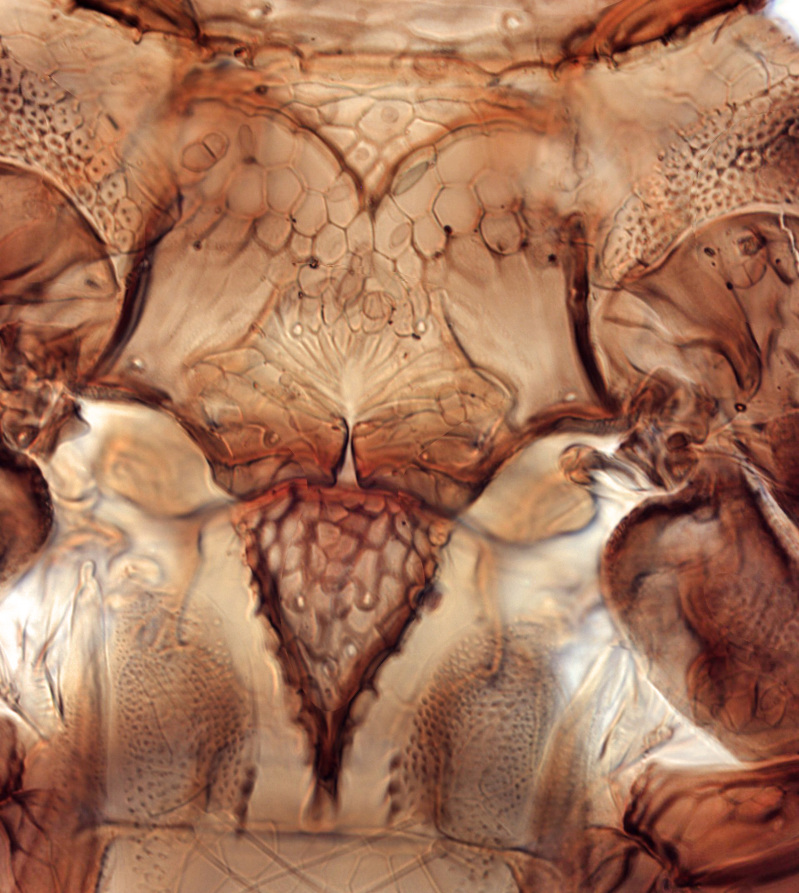
*Phibalothrips peringueyi* (Faure);

**Figure 3f.**
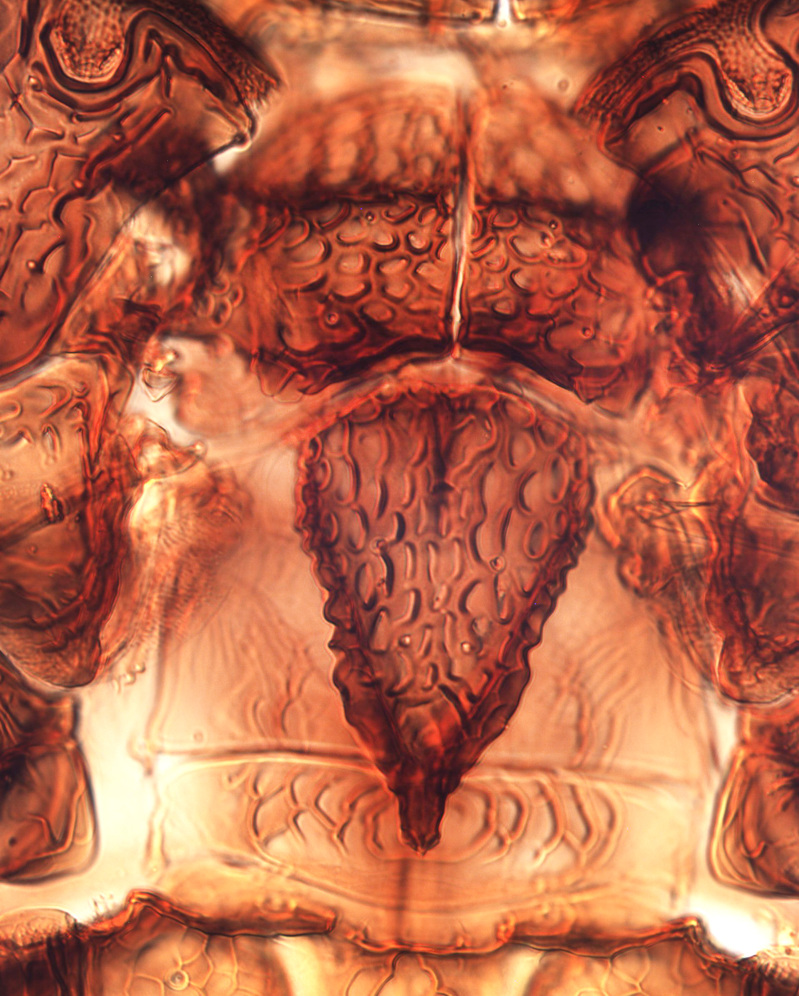
*Rhipiphorothrips cruentatus* Hood.

**Figure 4a.**
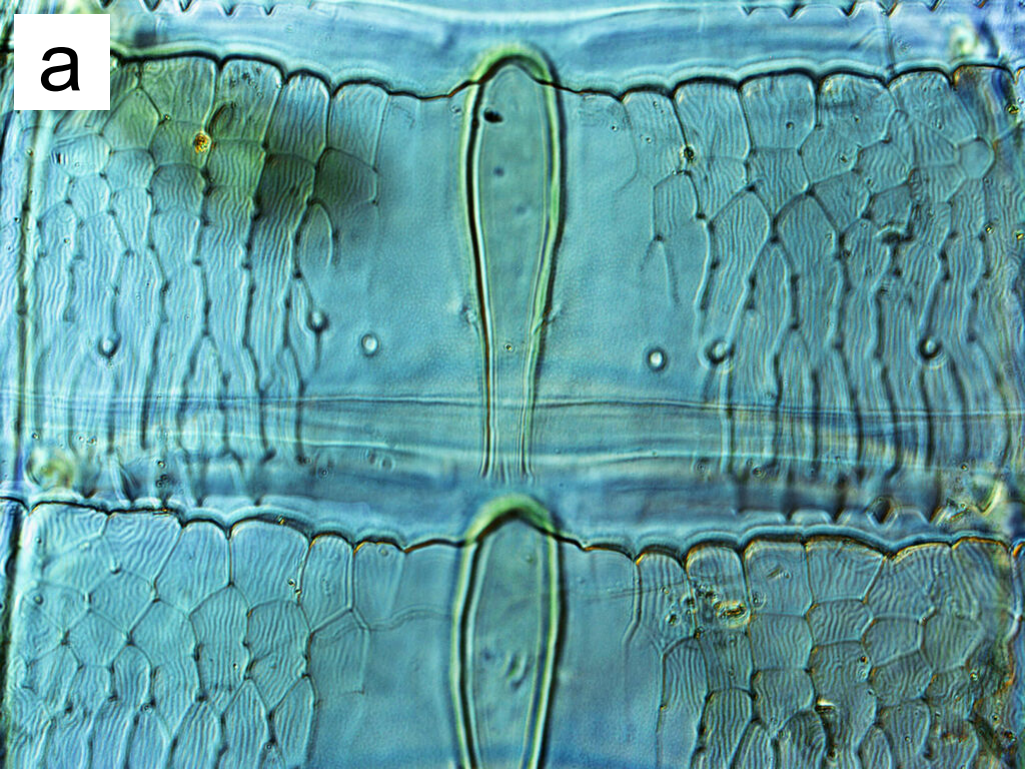
*Phibalothrips peringueyi* (Faure);

**Figure 4b.**
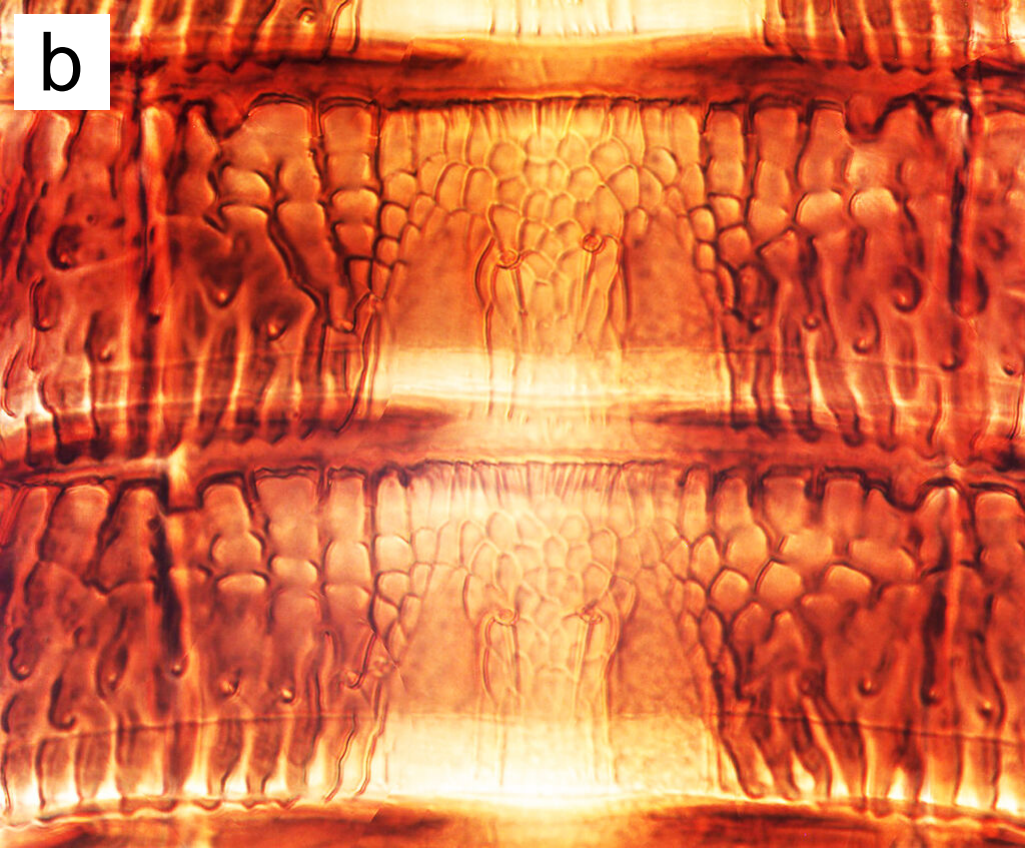
*Rhipiphorothrips cruentatus* Hood;

**Figure 4c.**
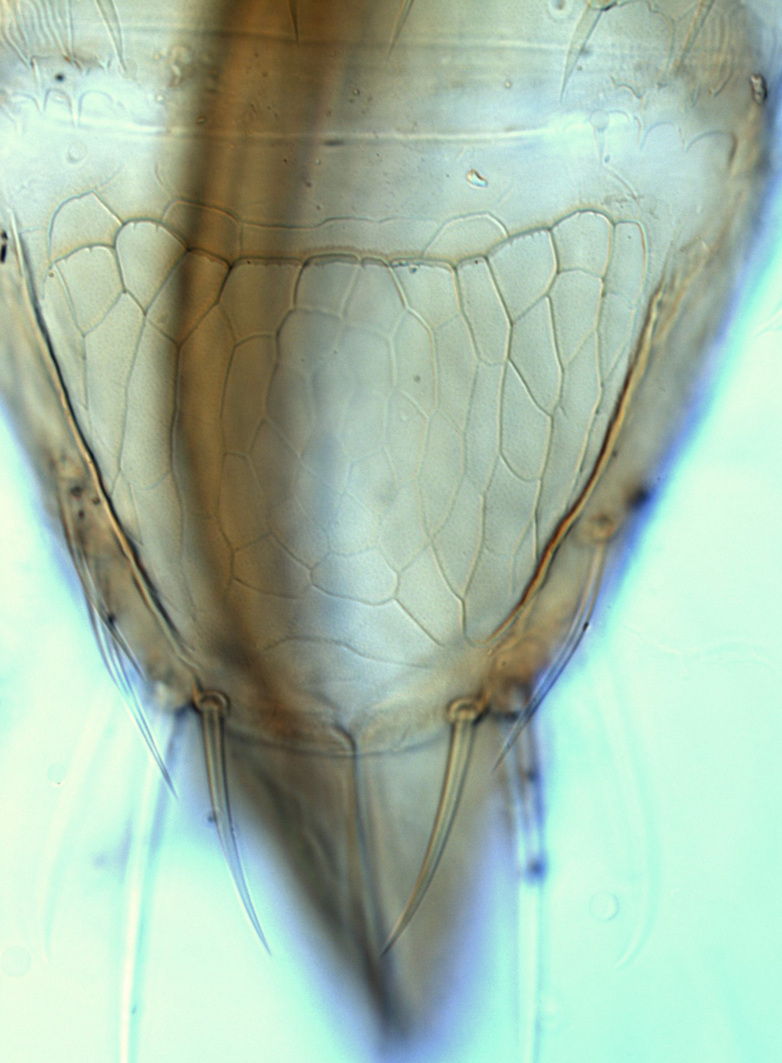
*Phibalothrips peringueyi* (Faure);

**Figure 4d.**
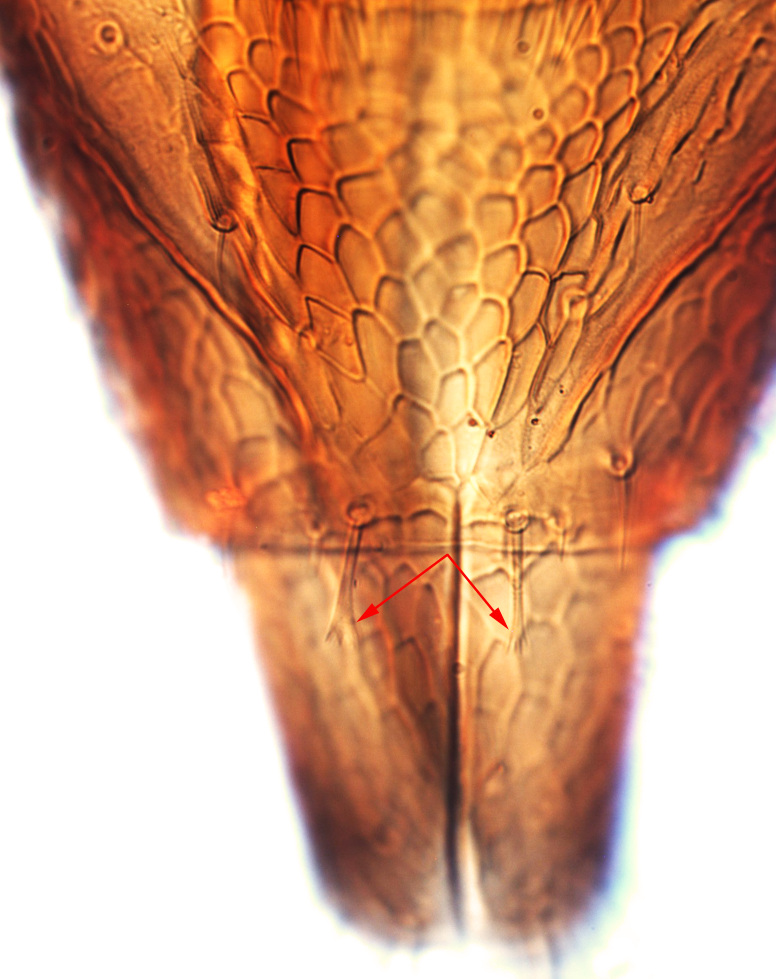
*Rhipiphorothrips cruentatus* Hood;

**Figure 4e.**
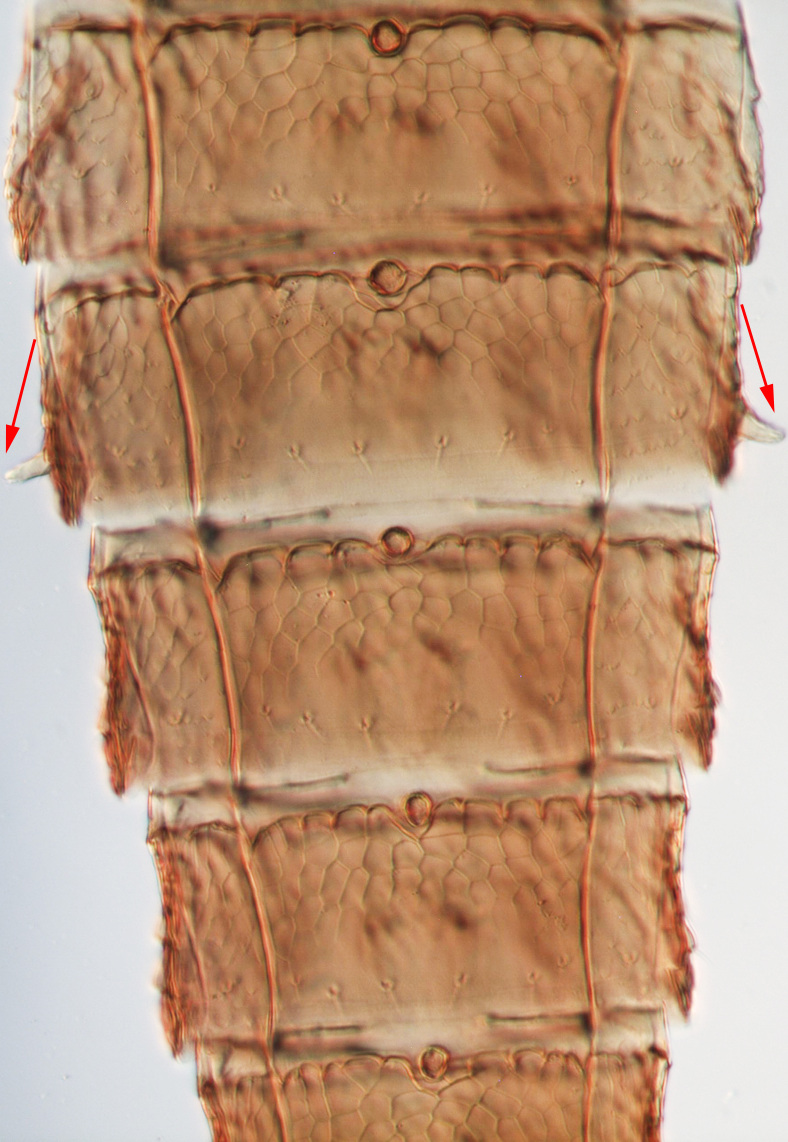
*Rhipiphorothrips cruentatus* Hood.

**Figure 5a.**
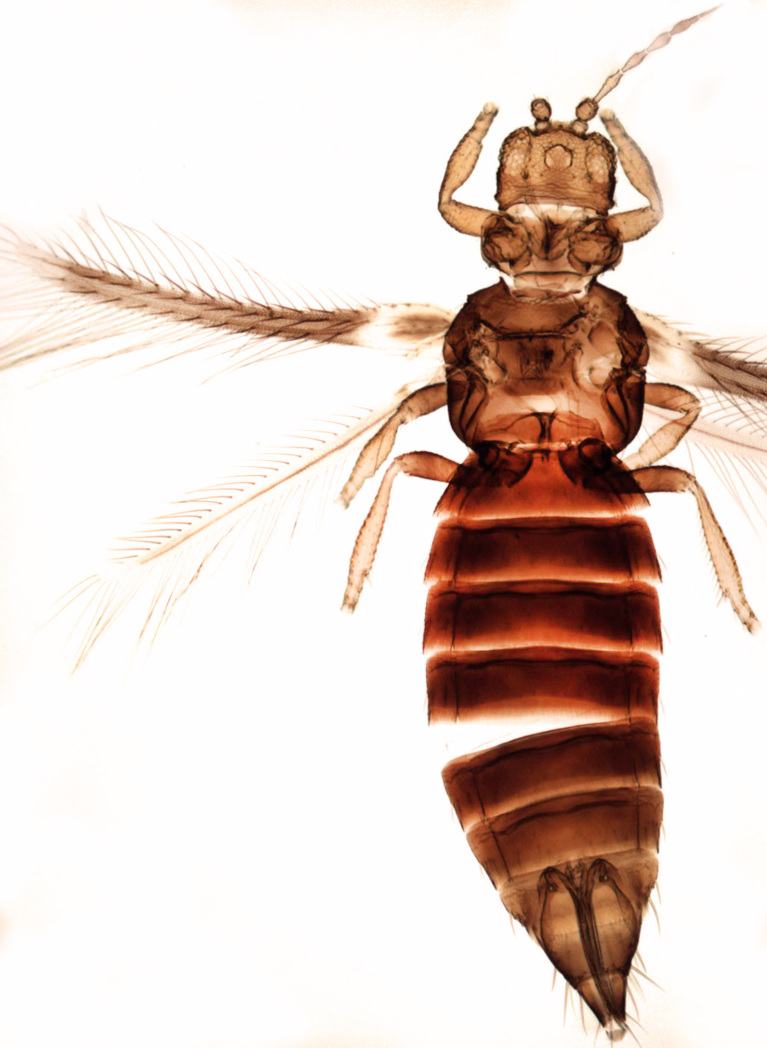
*Hercinothrips femoralis* Reuter;

**Figure 5b.**
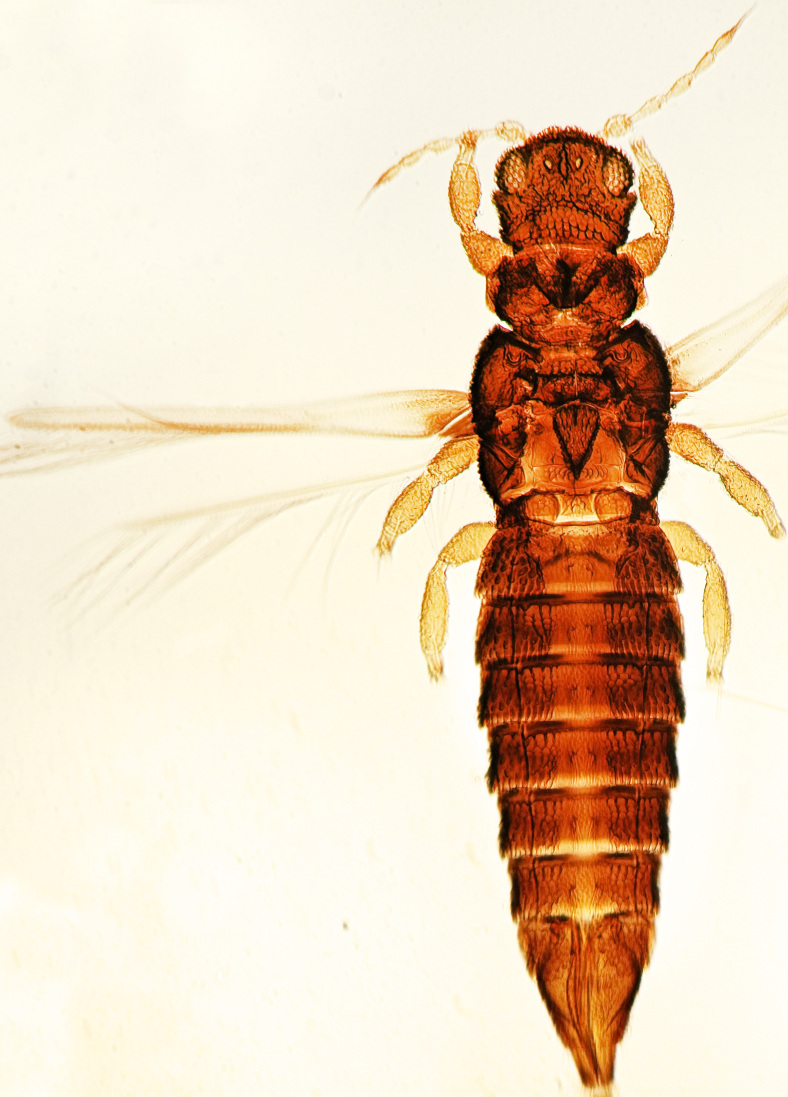
*Rhipiphorothrips cruentatus* Hood;

**Figure 5c.**
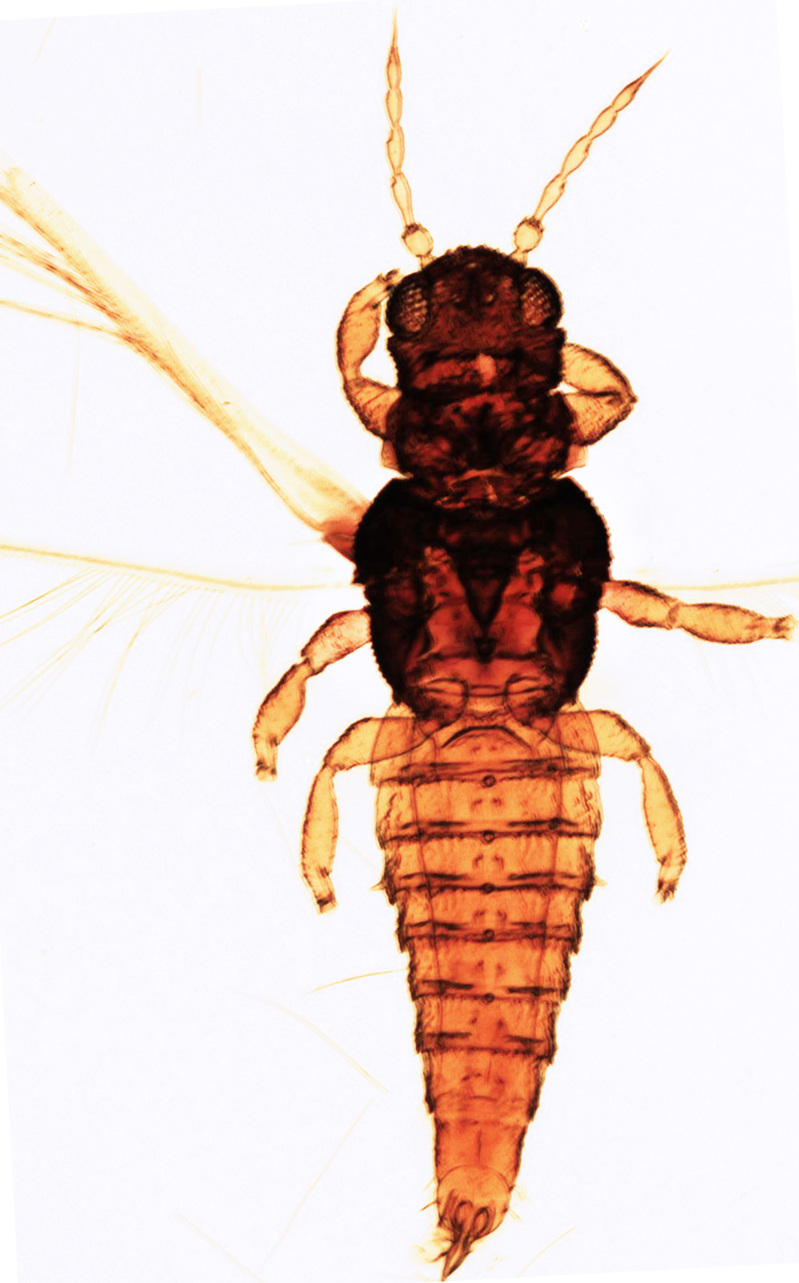
*Rhipiphorothrips cruentatus* Hood.

**Table 1.** A checklist of the Panchaetothripinae species from the Arabian Peninsula. The species with one asterisk (*) indicates a new record for Saudi Arabia and two asterisks (**) indicate the species was previously known from Saudi Arabia, but not recovered during this study.

**Species name**	**Bahrain**	**Iraq**	**Kuwait**	**Oman**	**Qatar**	**Saudi Arabia**	**United Arab Emirates**	**Yemen**
*Caliothrips sudanensis* (Bagnall & Cameron, 1932)	-	-	-	-	-	**l**	-	-
*Caliothrips indicus* (Bagnall, 1913)	-	**l**	-	-	-	-	-	-
*Caliothrips helini* Hood, 1940	-	-	-	-	-	**l**	-	**l**
*Caliothrips luckmanni* Wilson, 1975	-	-	-	-	-	**l**	-	-
*Caliothrips oneillae* Faure, 1962	-	-	-	-	-	**l**	-	-
*Caliothrips quadrifasciatus* (Girault, 1927)	-	-	-	-	-	**l**	**l**	**l**
*Phibalothrips peringueyi* (Faure, 1925)	-	-	-	-	-	**l**	-	-
*Rhipiphorothrips cruentatus* Hood, 1919*	-	-	-	**l**	-	**l**	-	-
*Retithrips syriacus* (Mayet, 1890)	-	**l**	-	-	-	**l**	**l**	**l**
*Hercinothrips femoralis* Reuter, 1891*	-	-	-	-	-	**l**	-	**l**
*Hercinothrips tenuis* Hartwig, 1948	-	-	-	-	-	-	-	**l**
*Heliothrips haemorrhoidalis* (Bouche, 1833) **	-	-	-	-	-	**l**	-	-
**Total**	**0**	**2**	**0**	**1**	**0**	**10**	**2**	**5**
